# Supplementary material for: Selective control of synaptically-connected circuit elements by all-optical synapses
Source: Commun Biol. 2022 Jan 11;5:33. doi: 10.1038/s42003-021-02981-7 (PMC8752598; doi:10.1038/s42003-021-02981-7)
Supplement: Supplementary file 3 — Description of Additional Supplementary Files [file 42003_2021_2981_MOESM3_ESM.pdf]

Description of Additional Supplementary Files

File name: Supplementary Data 1
Description: Modulation of activity by Interluminescence of excitatory and inhibitory opsin expressing postsynaptic neurons. Source data for Fig. 2d.

File name: Supplementary Data 2
Description: Communication via Interluminescence in co-cultured populations depends on intact synaptic connections. Source data for Fig. 3c.

File name: Supplementary Data 3
Description: Synaptic Blockers for isolating Interluminescence effects. Source data for Supplementary Fig. 4c.

File name: Supplementary Data 4
Description: Interluminescence elicits postsynaptic firing increase in the presence of synaptic blockers dependent on presynaptic neuronal activity. Source data for Figs. 4c and 4e.

File name: Supplementary Data 5.
Description: Interluminescence is dependent on presynaptic vesicle release. Source data for Fig. 5c.

File name: Supplementary Data 6
Description: Interluminescence is mediated by bioluminescence activation of the opsin. Source data for Figs. 6c and 6f.

File name: Supplementary Data 7
Description: Modulation of postsynaptic neural activity by Interluminescence in vivo. Source data for Figs. 7b, 7c, 7d, 7e and 7f.
