# Supplementary material for: Selective control of synaptically-connected circuit elements by all-optical synapses
Source: Commun Biol. 2022 Jan 11;5:33. doi: 10.1038/s42003-021-02981-7 (PMC8752598; doi:10.1038/s42003-021-02981-7)
Supplement: Supplementary file 11 — Reporting Summary [file 42003_2021_2981_MOESM11_ESM.pdf]

nature portfolio

Corresponding author(s): Ute Hochgeschwender

Last updated by author(s): Nov 30, 2021

Reporting Summary

Nature Portfolio wishes to improve the reproducibility of the work that we publish. This form provides structure for consistency and transparency in reporting. For further information on Nature Portfolio policies, see our Editorial Policies and the Editorial Policy Checklist.

Statistics

For all statistical analyses, confirm that the following items are present in the figure legend, table legend, main text, or Methods section.

n/a	Confirmed	
☐	☒	The exact sample size (*n*) for each experimental group/condition, given as a discrete number and unit of measurement
☐	☒	A statement on whether measurements were taken from distinct samples or whether the same sample was measured repeatedly
☐	☒	The statistical test(s) used AND whether they are one- or two-sided<br>*Only common tests should be described solely by name; describe more complex techniques in the Methods section.*
☒	☐	A description of all covariates tested
☒	☐	A description of any assumptions or corrections, such as tests of normality and adjustment for multiple comparisons
☐	☒	A full description of the statistical parameters including central tendency (e.g. means) or other basic estimates (e.g. regression coefficient) AND variation (e.g. standard deviation) or associated estimates of uncertainty (e.g. confidence intervals)
☐	☒	For null hypothesis testing, the test statistic (e.g. *F*, *t*, *r*) with confidence intervals, effect sizes, degrees of freedom and *P* value noted<br>*Give P values as exact values whenever suitable.*
☒	☐	For Bayesian analysis, information on the choice of priors and Markov chain Monte Carlo settings
☒	☐	For hierarchical and complex designs, identification of the appropriate level for tests and full reporting of outcomes
☒	☐	Estimates of effect sizes (e.g. Cohen's *d*, Pearson's *r*), indicating how they were calculated

Our web collection on statistics for biologists contains articles on many of the points above.

Software and code

Policy information about availability of computer code

Data collection	MC Rack software, Multichannel Systems, Germany, was used for in vitro MEA data acquisition.<br>Open Ephys acquisition board (http://www.open-ephys.org/) and Open Ephys GUI software were used for In vivo electro-physiological data acquisition.<br>Andor Solis data acquisition software (Andor Solis 64 bit, v4.31) was used for bioluminescence imaging data acquisition.
Data analysis	All MEA analysis was done offline with MC Rack software (Multichannel Systems; RRID: SCR_014955) and NeuroExplorer (RRID: SCR_001818).<br>Prism software (GraphPad 8.2.1; San Diego, CA) was used for in vitro MEA data figure generation and statistical analyses.<br>Offline analyses of both in vivo electrophysiological and bioluminescence imaging data were performed in Matlab R2020a (The Mathworks Inc.).

For manuscripts utilizing custom algorithms or software that are central to the research but not yet described in published literature, software must be made available to editors and reviewers. We strongly encourage code deposition in a community repository (e.g. GitHub). See the Nature Portfolio guidelines for submitting code & software for further information.

Data

Policy information about availability of data

All manuscripts must include a data availability statement. This statement should provide the following information, where applicable:

- Accession codes, unique identifiers, or web links for publicly available datasets
- A description of any restrictions on data availability
- For clinical datasets or third party data, please ensure that the statement adheres to our policy

All data generated and analysed during this study are included in this published article (and its supplementary information files). All raw data are available from the

corresponding authors upon reasonable request. The plasmid pAAV-hSyn-hPOMC1-26-sbGLuc-P2A-dTomato is available from Addgene (ID number 176704).

Field-specific reporting

Please select the one below that is the best fit for your research. If you are not sure, read the appropriate sections before making your selection.

- [x] Life sciences
- [ ] Behavioural & social sciences
- [ ] Ecological, evolutionary & environmental sciences

For a reference copy of the document with all sections, see nature.com/documents/nr-reporting-summary-flat.pdf

Life sciences study design

All studies must disclose on these points even when the disclosure is negative.

Sample size	For MEA experiments, pooled data was obtained from different electrodes (a) of the same culture, (b) from different cultures, and (c) over different days in vitro. All sample sizes are noted in the figure legends and accompanying text.<br>For the in vivo experiments, electrophysiology and image time-series data were recorded from three (3) experimental animals and three (3) control animals during a single recording session per animal after which the animal was euthanized.<br>Sample sizes were not predetermined based on statistical methods, but were chosen according to the standards of the field (at least three independent biological replicates for each condition), which gave sufficient statistics for the effect sizes of interest.
Data exclusions	Data from all noisy MEA electrodes were excluded.<br>In vivo imaging data for one of the Opsin (+) animals was excluded due to a software malfunction. Noisy electrode contacts in the in vivo electrophysiology were excluded from analyses, the method of exclusion is described in the in vivo methods section.
Replication	Reported results were consistently replicated across multiple experiments with all replicates generating similar results.
Randomization	The MEA experiments were randomized for different treatment conditions. For example, the MEA used for experimental condition was also tested for the control condition and vice versa, over different DIVs (days in vitro).<br>For the in vivo experiments, data were collected from experimental and control animals on randomly assigned days.
Blinding	Investigators were not blinded for the data collection and data analysis. Blinding during data collection was not needed because conditions were well controlled. Blinding during analysis was not needed because the results are quantitative and did not require subjective judgment or interpretation.

Reporting for specific materials, systems and methods

We require information from authors about some types of materials, experimental systems and methods used in many studies. Here, indicate whether each material, system or method listed is relevant to your study. If you are not sure if a list item applies to your research, read the appropriate section before selecting a response.

Materials & experimental systems

n/a	Involved in the study	
☐	☒	Antibodies
☒	☐	Eukaryotic cell lines
☒	☐	Palaeontology and archaeology
☐	☒	Animals and other organisms
☒	☐	Human research participants
☒	☐	Clinical data
☒	☐	Dual use research of concern

Methods

n/a	Involved in the study	
☒	☐	ChIP-seq
☒	☐	Flow cytometry
☒	☐	MRI-based neuroimaging

Antibodies

Antibodies used	rabbit polyclonal anti-Dopamine I> Hydroxylase (DJ,H) antibody (Millipore Sigma, AB 1585; diluted 1:2000) Donkey anti-Rabbit lgG H&L (Alexa Fluor 594; ab150076; diluted 1:500)
Validation	Anti-Dopamine I> Hydroxylase (DJ,H) antibody (Millipore Sigma, AB 1585) has been validated for specificity to stain only cells known to contain DBH and is abolished by preincubation with DBH (see company website, including literature references therein). Further, it has been validated for use in immunohistochemistry, Western blot, RIA, and inhibitory studies in vitro and in vivo, as stated on the MilliporeSigma product page.

Animals and other organisms

Policy information about studies involving animals; ARRIVE guidelines recommended for reporting animal research

Laboratory animals	PV-Cre mice (6 male; JAX stock #008069) aged 9 to 19 weeks, were used.
Wild animals	*Provide details on animals observed in or captured in the field; report species, sex and age where possible. Describe how animals were caught and transported and what happened to captive animals after the study (if killed, explain why and describe method; if released, say where and when) OR state that the study did not involve wild animals.*
Field-collected samples	*For laboratory work with field-collected samples, describe all relevant parameters such as housing, maintenance, temperature, photoperiod and end-of-experiment protocol OR state that the study did not involve samples collected from the field.*
Ethics oversight	The in vivo experiments and protocol were approved by the Institutional Animal Care and Use Committee at Brown University.

Note that full information on the approval of the study protocol must also be provided in the manuscript.
